# Spontaneous Hemoperitoneum from Bleeding of a Pedunculated Epiploic Appendage

**DOI:** 10.5334/jbsr.1530

**Published:** 2018-04-19

**Authors:** Bruno Coulier, Adrien Ramboux

**Affiliations:** 1 Clinique Saint-Luc, Bouge, BE

**Keywords:** hemoperitoneum, abdominal apoplexy, epiploic appendage

## Case Report

A 72-year-old patient was admitted in the emergency room with an 18-hour history of increasing abdominal pain in the right flank and iliac fossa. Laboratory tests showed anemia with hemoglobin at 97 g/l and renal failure with creatinine level at 21 mg/l.

**Figure 1:**
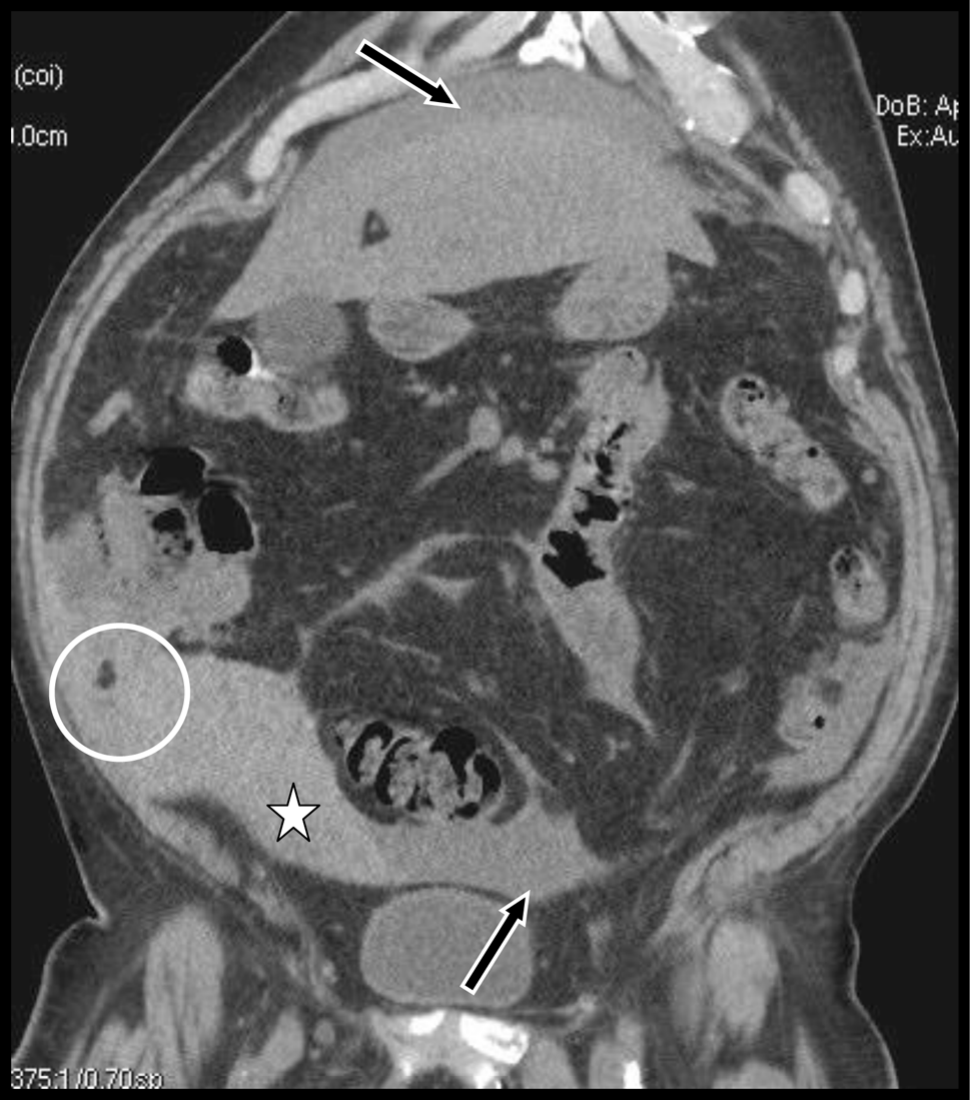
Coronal view of unenhanced abdominal CT shows diffuse ascites (black arrows) that appears spontaneously dense in the hypogastric area and in the right iliac fossa (white star) evocating hemoperitoneum. A small lipomatous masse is trapped in blood in the right iliac fossa (white circle).

**Figure 2:**
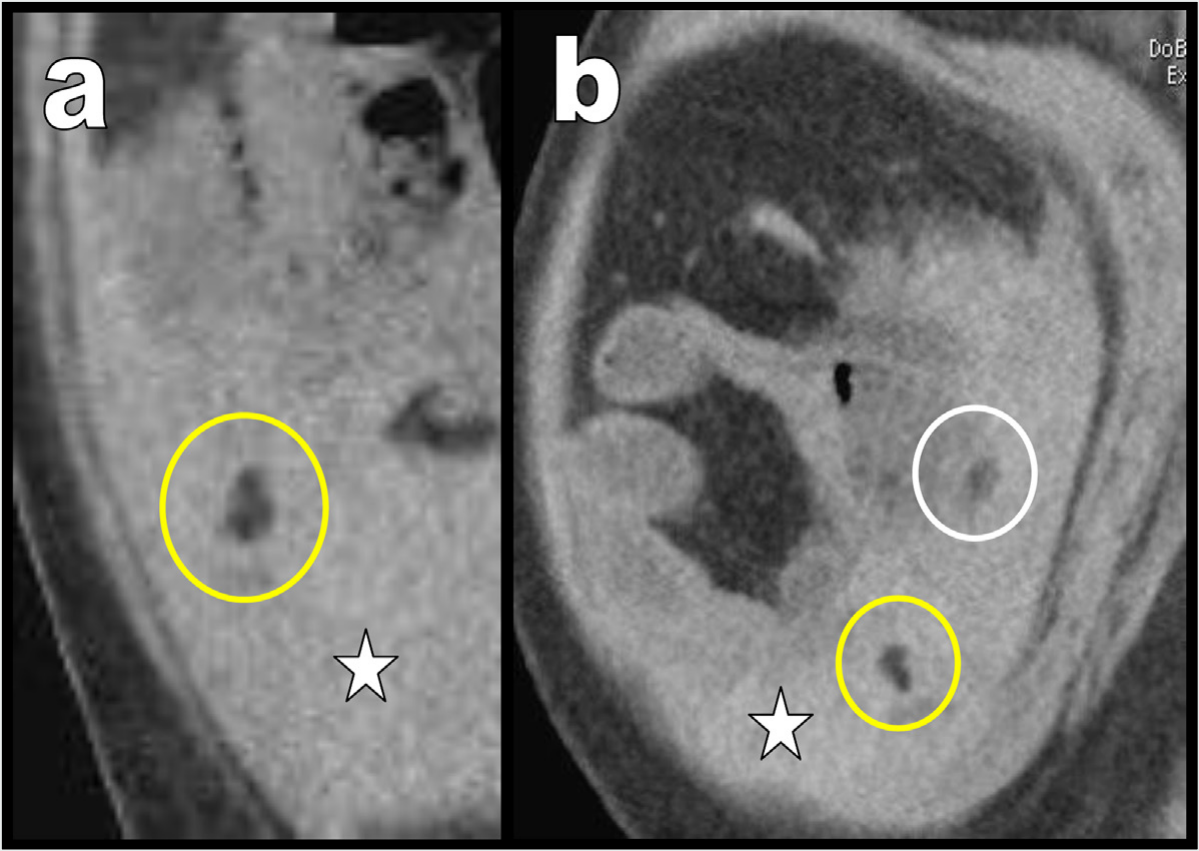
Two coronal CT oblique views of the right iliac fossa (**a** & **b**) showing small lipomatous masses (yellow and white circles) trapped in fresh blood (white star).

**Figure 3:**
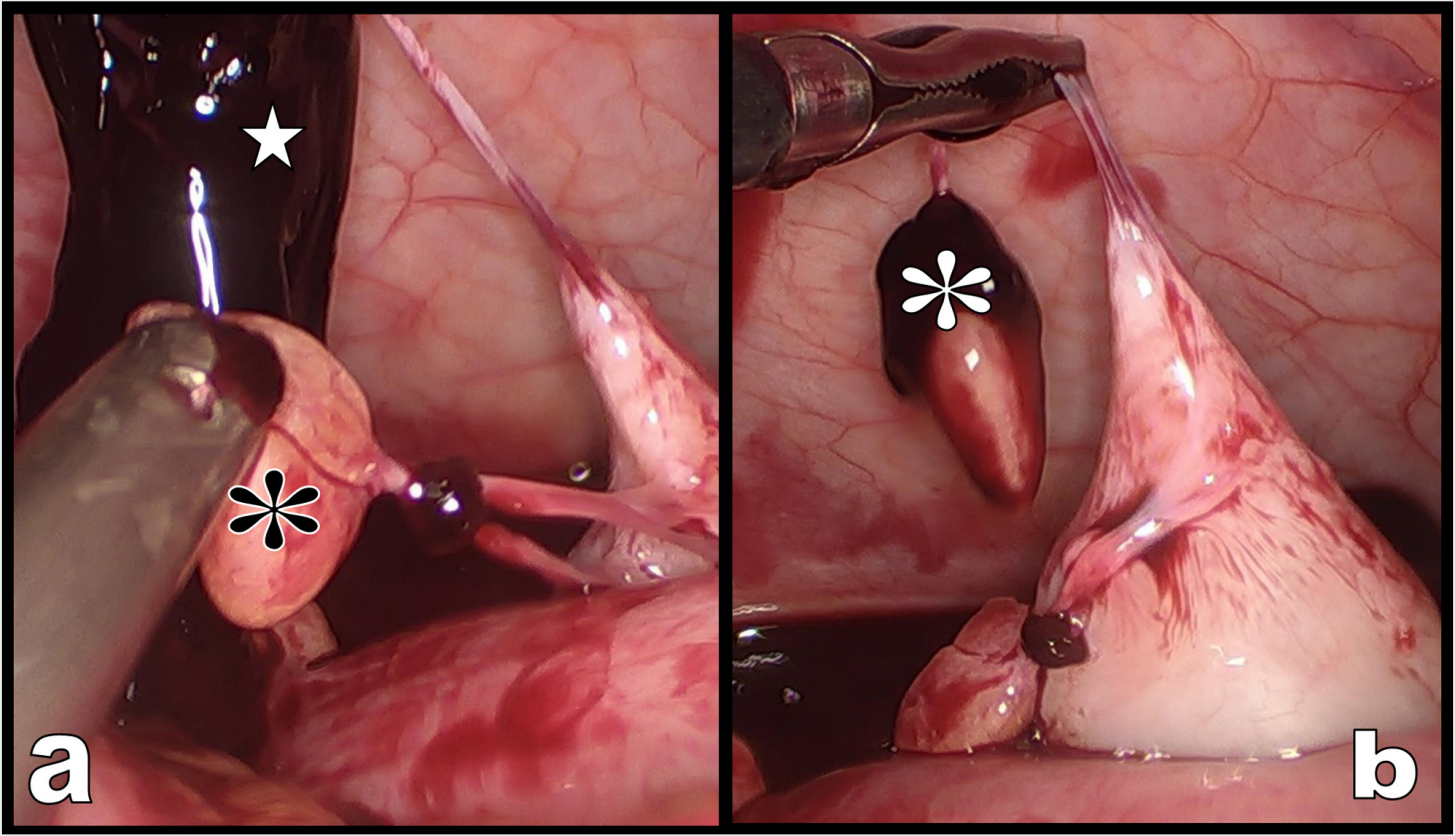
Emergency coelioscopy (**a** & **b**) confirms massive hemoperitoneum. Two long pedunculated lipomatous appendages (white and black asterisks) are found. One of them was bleeding and was wrapped in a large fresh blood clot (white star on b).

Unenhanced abdominal computed tomography (CT) showed signs of cirrhotic dysmorphism of the liver (not illustrated) and diffuse ascites (black arrows on coronal view, Figure 1) that appeared spontaneously dense in the hypogastric area and in the right iliac fossa (white star) suggesting hemoperitoneum. Two small lipomatous masses were visible being trapped in blood in the right iliac fossa (yellow and white circles on Figures 1 and 2). Emergency coelioscopy (Figure 3) confirmed more than one liter of blood in the peritoneal cavity. Exploration of the right iliac fossa revealed two long pedunculated lipomatous appendages (white and black asterisks on Figure 3). One of them was bleeding and was wrapped in a large blood clot (white star). A complete revision of the entire abdominal cavity didn’t reveal any other cause of bleeding. Traction and/or torsion of the pedunculated lipomatous appendage with secondary bleeding was diagnosed as the cause of hemoperitoneum. The lipomatous appendages were clipped and resected. The post-operative period was uneventful.

## Comment

In clinical practice, most cases of hemoperitoneum are the result of numerous well-documented causes comprising open or blunt abdominal trauma, bleeding visceral malignancies, gynecologic diseases such as ectopic pregnancy or inflammatory eroding processes such as pancreatitis and/or pseudocyst.

Consequently, residual cases of spontaneous intra-abdominal haemorrhage (or abdominal apoplexy) are exceedingly rare events related to bleeding of small secondary abdominal arteries or veins.

Only rare cases of spontaneous omental haemorrhage have been reported. They are usually preceded by trauma or occur in the context of adhesions, torsion, neoplasms, vasculitis, varices, anticoagulation or ectopic omental pregnancy [1].

The implication of epiploic appendages in acute abdominal emergencies is greatly dominated by appendagitis due to acute torsion or infarction. The vast majority of epiploic appendagitis are self-limiting events with only localized peritoneal inflammatory reaction and with characteristic imaging features.

To the best of our knowledge a spontaneous hemoperitoneum related to bleeding of an epiploic appendage has never been reported before. In our case the presence of a very long peduncle may have favoured a possible traction with tearing of a small vessel of the epiploic appendage.
